# Genome-Wide Association Mapping of Loci Associated with Plant Growth and Forage Production under Salt Stress in Alfalfa (*Medicago sativa* L.)

**DOI:** 10.3389/fpls.2017.00853

**Published:** 2017-05-24

**Authors:** Xiang-Ping Liu, Long-Xi Yu

**Affiliations:** ^1^United States Department of Agriculture-Agricultural Research Service, Plant Germplasm Introduction and Testing ResearchProsser, WA, United States; ^2^College of Animal Science and Veterinary Medicine, Heilongjiang Bayi Agricultural UniversityDaqing, China

**Keywords:** GWAS, alfalfa, SNP, linkage disequilibrium, salt tolerance

## Abstract

Salinity tolerance is highly desirable to sustain alfalfa production in marginal lands that have been rendered saline. In this study, we used a diverse panel of 198 alfalfa accessions for mapping loci associated with plant growth and forage production under salt stress using genome-wide association studies (GWAS). The plants were genotyped using genotyping-by-sequencing (GBS). A greenhouse procedure was used for phenotyping four agronomic and physiological traits affected by salt stress, including dry weight (DW), plant height (PH), leaf chlorophyll content (LCC), and stomatal conductance (SC). For each trait, a stress susceptibility index (SSI) was used to evaluate plant performance under stressed and non-stressed conditions. Marker-trait association identified a total of 42 markers significantly associated with salt tolerance. They were located on all chromosomes except chromosome 2 based on the alignment of their flanking sequences to the reference genome (*Medicago truncatula*). Of those identified, 13 were associated with multiple traits. Several loci identified in the present study were also identified in previous reports. BLAST search revealed that 19 putative candidate genes linked to 24 significant markers. Among them, B3 DNA-binding protein, Thiaminepyrophosphokinase and IQ calmodulin-binding motif protein were identified among multiple traits in the present and previous studies. With further investigation, these markers and candidates would be useful for developing markers for marker-assisted selection in breeding programs to improve alfalfa cultivars with enhanced tolerance to salt stress.

## Introduction

Salinity affects crop production in approximately 830 million hectares worldwide and the acreage is increasing in the arid and semi-arid regions where saline water is used for irrigation (Rengasamy, 2006). Alfalfa (*Medicago sativa* L.) is the most widely grown forage crop with a high yield and nutritive value. Soil salinity causes a significant reduction in alfalfa yield. Alfalfa production decreases when soil salinity is above a threshold of 2.0 ds m^−1^ (~22 mM NaCl) (Johnson et al., 1992). The development of salt-tolerant alfalfa varieties is highly desirable to sustain alfalfa production in marginal lands that have been rendered saline.

Physiological responses of plants to salinity can be partitioned to two phases: a rapid, osmotic phase that reduces or inhibits growth of young leaves and a slower, ionic phase that accelerates senescence of mature leaves (Munns and Tester, 2008). Affenzeller et al. (2009) found that the ionic of salt stress led to the activation of the endonuclease resulting in programmed cell death that plays an important role in mediating plant adaptive response to salinity. Numerous reports have shown that the chlorophyll content declined under salt stress in leaves (Hernandez et al., 1999; Agastian et al., 2000; Wang and Nil, 2000). It has been reported that stomatal conductance under salt stress is reduced due to changes in cell anatomy (James et al., 2002). Genetic variability of salt tolerance exists within populations and it has been used for selection of salt-tolerant alfalfa (Kapulnik et al., 1989; Johnson et al., 1992). Physiological traits related to salt tolerance have also been investigated and used as factors for improving alfalfa salt tolerance (Ashraf et al., 1986; Monirifar and Barghi, 2009). Although efforts have been made on the selection of salt-tolerant alfalfa using traditional breeding (Smith et al., 1994; Scasta et al., 2012) and genetic engineering (Liu et al., 2011; Zhang and Wang, 2015), few salt-tolerant cultivars have been released.

The traditional breeding strategy for improving alfalfa with salt tolerance is slow due to the genetic complexity of this autotetraploid species. One way to improve breeding selection efficiency is to identify genetic loci associated with salt tolerance and developing diagnostic markers closely linked to the tolerance loci for marker-assisted selection (Lande and Thompson, 1990). Genetic linkage maps have been constructed in both diploid (*M. truncatula*) and tetraploid alfalfa (*M. sativa*) using various marker platforms including restriction fragment length polymorphism (RFLP), simple sequence repeat (SSR), random amplified polymorphic DNA (RAPD), and amplified fragment length polymorphism (AFLP) markers. QTLs associated with biomass (Robins et al., 2007b), morphological traits (Robins et al., 2007a), winter hardiness, persistence (Brouwer et al., 2000; Alarcón-Zúñiga et al., 2004), self-fertility (Robins and Brummer, 2010), Stagonospora root and crown rot resistance (Musial et al., 2007), anthracnose resistance (Mackie et al., 2007) and aluminum tolerance (Khu et al., 2010) have been identified in alfalfa. QTLs associated with salt tolerance have been identified in the model legume, *M. truncatula* and mapped to all chromosomes except chromosomes 5 and 6 (Arraouadi et al., 2011, 2012). In addition, a number of salt-responsive genes have recently been identified and characterized in alfalfa (Jin et al., 2010; Soto et al., 2011).

Yield under abiotic stresses such as drought and high salinity is most likely under the control of multiple genes and interact with environmental factors. Identification of loci that contribute to variation in such complex traits is a primary challenge in plant breeding and population genetics. Genome-wide association studies (GWAS) provide an integrated framework that merges a QTL mapping approach with high-throughput next-generation sequencing methodology to map stress tolerance loci. This framework provides a statistical basis for analyzing marker-trait association using linkage disequilibrium. It has been used with success in both humans (Cardon and Abecasis, 2003; Manolio et al., 2008) and plants. In plants, GWAS have been applied to Arabidopsis (Atwell et al., 2010), rice (Huang et al., 2010), wheat (Tadesse et al., 2015), soybean (Zhang J. et al., 2015; Patil et al., 2016), maize (Liu et al., 2016; Olukolu et al., 2016), and *M. truncatula* (Stanton-Geddes et al., 2013; Kang et al., 2015). In alfalfa, QTL related to disease resistance (Liu et al., 2014), drought tolerance (Zhang T. et al., 2015) and forage quality (Wang et al., 2016) have been identified using GWAS. Most recently, we used GWAS and identified salt tolerance loci during germination in alfalfa (Yu et al., 2016).

To better understand the genetic base of salt tolerance in alfalfa, in the present study, we performed GWAS on traits related to plant growth and forage production under salt stress in the same panel of alfalfa accessions previously used for mapping loci associated with drought tolerance (Zhang T. et al., 2015) and salt tolerance during germination (Yu et al., 2016). We then compared SNPs significantly associated with salt tolerance within this study and between the present and previous studies. Our goal is to identify loci linked to forage yield under salt stress and understand the genetic base by which alfalfa germination, growth and forage production are affected by salt stress.

## Materials and methods

### Plant materials and experimental conditions

A total of 198 accessions selected from the USDA-ARS National Plant Germplasm System (NPGS) alfalfa collection (http://www.ars-grin/npgs.gov) were used and previously described (Zhang J. et al., 2015; Yu et al., 2016). Single representative plants were grown and clonally propagated in the USDA greenhouse in Prosser, WA. The propagated plants were then transplanted individually into 4-inch square pots filled with commercial potting soil (Pro-Mix) and grown in a greenhouse at 22°C and 40% relative humidity. The light density was approximately 200 μmol m^−2^s^−1^ (16 h/day). To ensure plants were approximately the same size, the plants were trimmed to 5 cm length once they began to flower. Fertilizer (Technigro® 20-20-20 All Purpose, Sun Gro Horticulture Canada Ltd.) was applied as needed.

The clonally propagated plants from each accession were divided into two groups: Group 1 for salt stress and group 2 for non-treatment control. Each group contained 3 replications with a complete randomized design. The plants in both groups were watered regularly with tap water until 21 days after transplanting. After that, group 1 for salt treatment were watered with saline water containing 4.68 g/L (equivalent to 80 mM) NaCl 4 weeks until harvesting. Group 2 for control were continuously watered with tape-water throughout the experiment.

### Measurement of traits

Before harvesting, the plant height (PH) of each plant was measured from the soil surface to the top of the main plant stem using a centimeter ruler.

The above-ground biomass was harvested and the dry weight (DW) was measured after drying at 80°C for 2 days in the oven.

The stomatal conductance (SC) of the leaf was measured using an SC-1 Leaf Porometer (Decagon Devices, Inc., Pullman, WA) on the leaf lamina of fully expanded leaves (avoiding the mid-rib and edges). Two measurements were performed on different leaves of the same plant. The mean value was used for evaluating the stomatal conductance of each plant.

Leaf chlorophyll content (LCC) was measured using a Leaf Chl Meter (FT Green LLC, Wilmington, DE) on the leaf lamina of fully expanded leaves (avoiding the mid-rib and edges). The measurements were performed on two different leaves of the same plant. The mean value was used for evaluating LCC of each plant.

The soil electrical conductivity (Soil EC) was measured with a ProCheck (Decagon Devices, Inc., Pullman, WA) to control the actual salt concentration imposed on each plant. The measurements were performed on two different positions within the soil of the same pot.

### Phenotypic data analysis

To evaluate plant response to salt stress, a stress susceptibility index (SSI) was used for all traits tested. It is calculated according to Fischer and Maurer (1978) as follows:

$$\begin{array}{l} \text{SSI}{=}^{\frac{Ys}{Yn}}{/}_{\frac{Ms}{Mn}} \end{array}$$

Where, *Ys* = performance of a specific accession under stress; *Yn* = performance of the same accession without stress; *Ms* and *Mn* are the mean values of performance overall of accessions in the given test under stress and non-stress conditions, respectively.

A statistical analysis was conducted for each trait. The equality of variance and means were analyzed by using ANOVA. Meanwhile, a normality test was performed for each trait using the Kolmogorov-Smirnov tests. Pearson correlation was calculated between traits using trait means. All analyses were conducted using SPSS 19 (http://www.ibm.com/software/analytics/spss/).

Broad-sense heritability (*H*^2^) was calculated using the formula *H*^2^ = *V*_*G*_/(*V*_*G*_+*V*_*E*_), where *V*_*G*_ and *V*_*E*_ were genotypic variance and environmental variance, respectively. The genotypic coefficient of variation (GCV) was calculated using the formula of

$$\begin{array}{l} GVC\left(\%\right)={\sqrt{V}}_{G}/X\times 100 \end{array}$$

Where, *X* is average of each trait of the whole association panel (Ahsan et al., 2015).

### SNP genotyping

The same SNP data as described in our previous report (Yu et al., 2016) were used in the present study. Briefly, DNA was extracted from young leaves of the original plants of 198 genotypes using the Qiagen DNeasy 96 Plant kit (Qiagen, CA), according to the manufacturer's protocol. A Nanodrop 1000 spectrophotometer was used for quantifying DNA at 260 nm absorbance (Thermo Scientific, http://www.thermoscientific.com). GBS libraries were prepared by digestion of DNA with the EcoT221 restriction enzyme and ligated to barcode and common adaptors as described by Elshire et al. (2011). They were placed in two lanes of an Illumina Hi-Seq2000 instrument for sequencing using 100-base single-end at Cornell University Sequencing facility (Ithaca, NY).

A GBS pipeline consisting of a sequence of software tools was used for genotype calling according to Glaubitz et al. (2014). The detailed procedure was described in Zhang T. et al. (2015).

The GBS sequences were submitted to the NCBI Sequence Read Archive with bioproject ID: PRJNA287263 and biosample accession numbers: AMN03779142–SAMN03779330.

### Genome-wide association study

SNPs were further filtered with a 5% cutoff value for minor allele frequency and 25% for missing data. The remaining 4,653 SNPs were subjected for GWAS. The phenotypic data of plant height, leaf chlorophyll content, stomatal conductance, and dry weight were calculated with SSI and the resulting values were used for GWAS. A mixed linear model (MLM, Q+K model) was used to analyze the marker-trait association using TASSEL (http://www.maizegenetics.net/tassel). A false discovery rate (FDR) of 0.05 was used for multiple test correction to determine significant association according to Benjamini and Hochberg (1995). The FDR has similar function as Bonferroni correction and has been used as the threshold for determining the significance in association mapping.

## Results

### Phenotypic variation and correlation between characterized traits

To evaluate phenotypic variation, we performed a statistical analysis for the four traits using ANOVA. Significant difference was found in each trait among the 198 accessions under both salt stress and control conditions (Table 1). A wide range of coefficient of variation (CV) was observed. The highest CV (82.2%) was found in the dry weight of stressed plants while the LCC of control plants showed lowest CV (18.2%). In addition, CV increased in dry weight, leaf chlorophyll content, stomatal conductance (SC)] and decreased in Soil EC (Table 1). The average values of all traits were much lower in salt stressed plants than those of control plants. The average dry weight and plant height of stressed plants decreased by 43.84 and 17.43%, respectively, compared to the control plants (Table 1, “DW and PH”). For the physiological traits, the average value of stomatal conductance of stressed plants decreased by more than half (57.68%) compared to the control plants (Table 1, “SC”), whereas relatively smaller effect was found on leaf chlorophyll content compared to other traits (Table 1, “LCC”). In contrast, the average value of the soil electrical conductivity was much higher in the stressed (2.08) than that in the control (0.2), indicating salt concentration was much higher in the stressed pots than that in the control (Table 1, “Soil EC”). The broad-sense heritability (*H*^2^) of the traits ranged from 8.37% (SC) to 46.08% (DW). A similar trend was observed for the GCV which ranged from 8.36% (SC) to 81.26% (DW).

**Table 1:** **Phenotypic variation for four traits including dry weight (DW), plant height (PH), leaf relative chlorophyll content (LCC), stomatal conductance (SC), and soil electrical conductivity (Soil EC) among 198 accessions**.

**Trait**		**Max**.	**Min**.	**Ave**.	**Change (%)**	**CV**	***F-*value**	**Sig**.	***H*^2^(%)**	**GCV (%)**
DW (g)	Control	2.37	0.03	0.73	43.84	72.6%	3.24	0.000^***^	42.19	81.26
	Salt	1.52	0.00	0.41		82.2%	3.79	0.000^***^	46.08	29.75
PH (cm)	Control	49.42	5.26	21.46	17.43	40.3%	2.39	0.000^***^	31.71	26.66
	Salt	40.00	5.45	17.72		39.7%	2.32	0.000^***^	30.91	17.58
LCC^#^	Control	70.1	19.2	51.21	5.17	18.2%	1.84	0.000^***^	21.79	8.81
	Salt	74.3	17.8	48.56		26.3%	2.65	0.000^***^	33.78	13.96
SC (mmol/m^2^/s)	Control	1005.3	26.5	414.91	57.68	49.4%	1.73	0.005^**^	18.86	49.79
	Salt	702.5	11.2	175.57		68.7%	1.38	0.005^**^	8.37	8.36
Soil EC (dS/m)	Control	0.84	0.01	0.20	90.38	57.4%	1.21	0.063	–	–
	Salt	4.60	0.29	2.08		33.2%	1.36	0.007^**^	–	–

CV, coefficient of variation. H^2^, broad-sense heritability based on accession mean. GCV, genetic variation coefficient.

“**” and “***” represents a significant difference at P < 0.01 and < 0.001, respectively.

“#″ *the unit of leaf chlorophyll is at Leaf value*.

**Figure 1:**
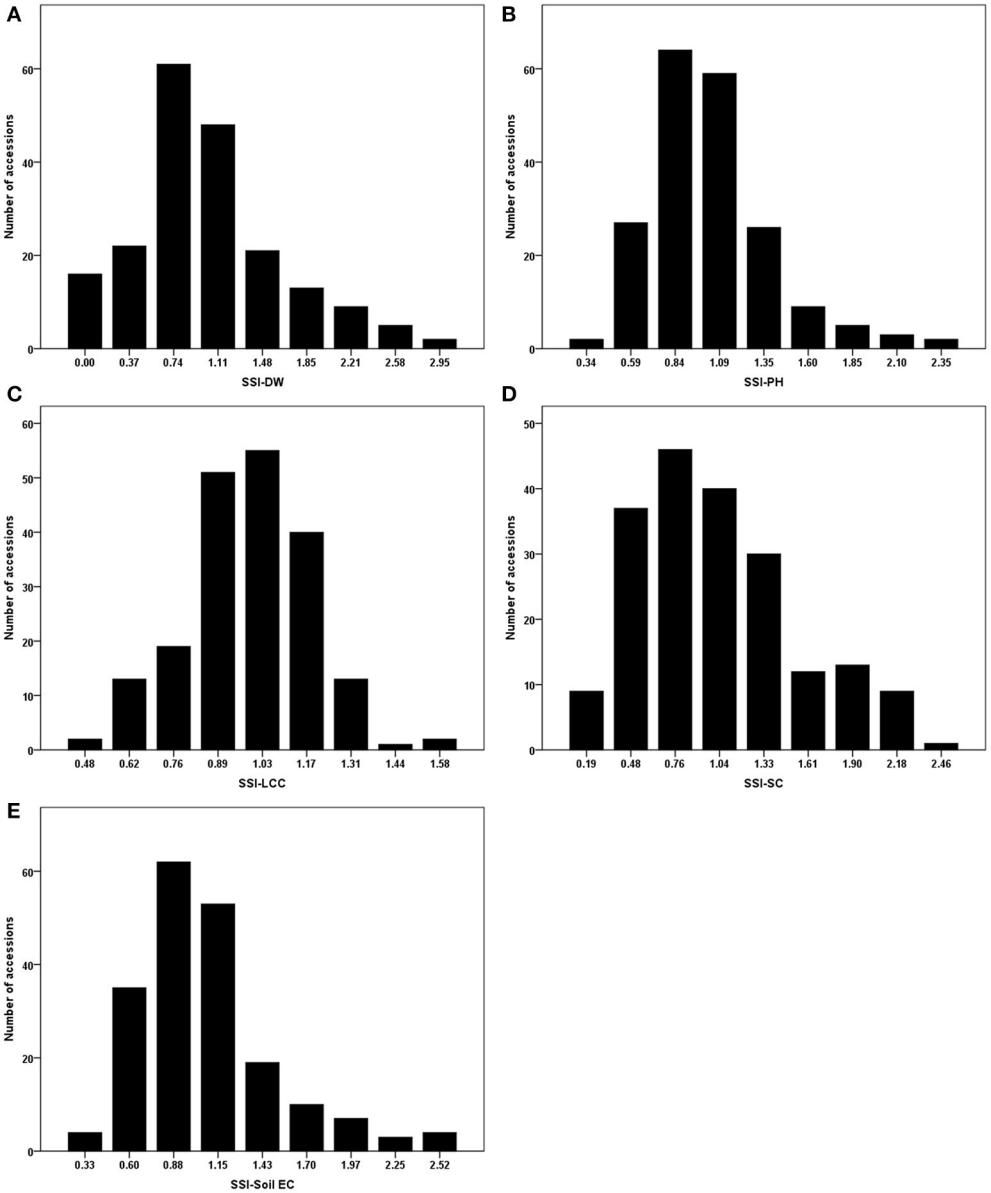
**Phenotypic evaluations of stress susceptibility indexes (SSI) for measured traits**. **(A)** SSI-DW, SSI for dry weight; **(B)** SSI-PH, SSI for plant height; **(C)** SSI-LCC, SSI for leaf relative chlorophyll content; **(D)** SSI-SC, SSI for stomatal conductance; **(E)** SSI-Soil EC, SSI for soil electrical conductivity.

As mentioned in the section of Materials and Methods, stress susceptibility index (SSI) calculates the ratio of yield reduction due to stress in a given genotype as compared to the mean reduction over all genotypes in a given test. The use of SSI is an improved measurement over the simple expression of a trait under stress as a percentage of the trait measured under non-stress conditions. The SSI presents not only phenotypic variation under stress condition but also takes into account of the genetic variability under non-stress condition. We therefore used the resulting SSI values for further analyses. Using these values, normal distributions were obtained for dry weight, plant height, leaf chlorophyll content, stomatal conductance and Soil EC (Figure 1). Correlations among the traits were also analyzed, and their correlation coefficients (r) were presented in Table 2. Positive correlations were obtained between all traits except Soil EC. Among those, the highest correlation was obtained between SSI-DW and SSI-PH (*r* = 0.604), while the lowest correlation was detected between SSI-DW and SSI-SC (*r* = 0.169). As expected, the soil electrical conductivity was negatively correlated with all other traits (Table 2).

**Table 2:** **Correlation coefficients (r) of SSI-DW, SSI-PH, SSI-LCC, and SSI-SC collected from 198 accessions**.

	**SSI-LCC**	**SSI-PH**	**SSI-SC**	**SSI-DW**	**SSI-Soil EC**
SSI-LCC	1	0.516^**^	0.351^**^	0.478^**^	−0.020
SSI-PH		1	0.261^**^	0.604^**^	−0.208^**^
SSI-SC			1	0.169^*^	−0.196^**^
SSI-DW				1	−0.039
SSI-Soil EC					1

** Significant at P < 0.01,

* *Significant at P < 0.05. SSI-LCC, for leaf relative chlorophyll content; SSI-PH, for plant height; SSI-SC, for stomatal conductance; SSI-DW, for dry weight, SSI-Soil EC for soil electrical conductivity*.

**Figure 2:**
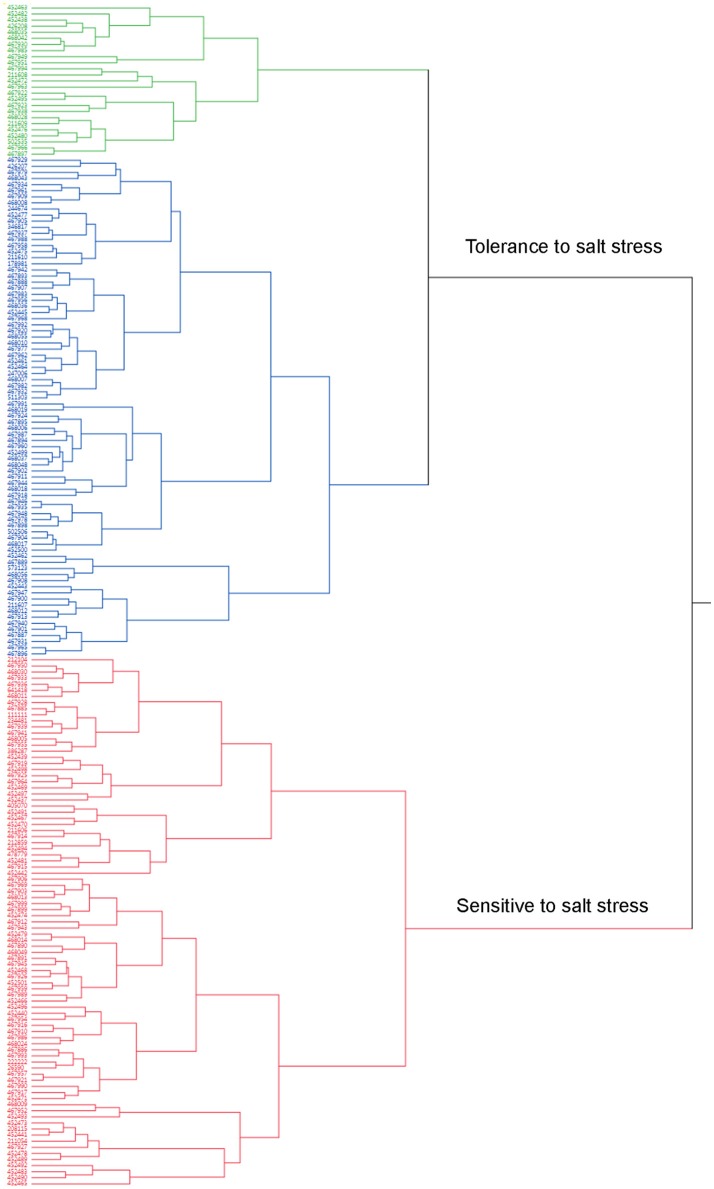
**Dendrogram of phenotypic variations of salt tolerance in 198 accessions**. Mean values of all traits evaluated in the present study were used for cluster analysis using the hierarchical cluster program built in the JMP13 software (SAS, Cary, NC). Accessions (PIs) were clustered into 2 clusters based on their responses to salt stress (Table S1). Cluster 1 contains 114 accessions (Blue and green colors) with resistant (Green colored) or moderate resistance (Blue) to salt stress. Cluster 2 contains 85 accessions (Red color) and most of them are sensitive to salt stress.

A cluster analysis was also carried out using JMP13 (SAS, Cary, NC). Phenotypic values of all traits were used for hierarchical cluster analysis (Table S1). Two clusters were obtained according to the cluster analysis (Figure 2). Cluster 1 contained 114 accessions and they were tolerant (Figure 2, green) to moderate tolerant (Figure 2, blue) to salt stress based on the phenotypic evaluation (Table S1). Cluster 2 contained 85 genotypes and most of them were sensitive to salt stress (Figure 2, red). Most of accessions in the tolerance cluster were from US and Canada. Five additional accessions (PIs 211606, 211608, 211609, 212104, and 244674) from Afghanistan were also clustered in the resistance group.

### Marker-trait association

**Figure 3:**
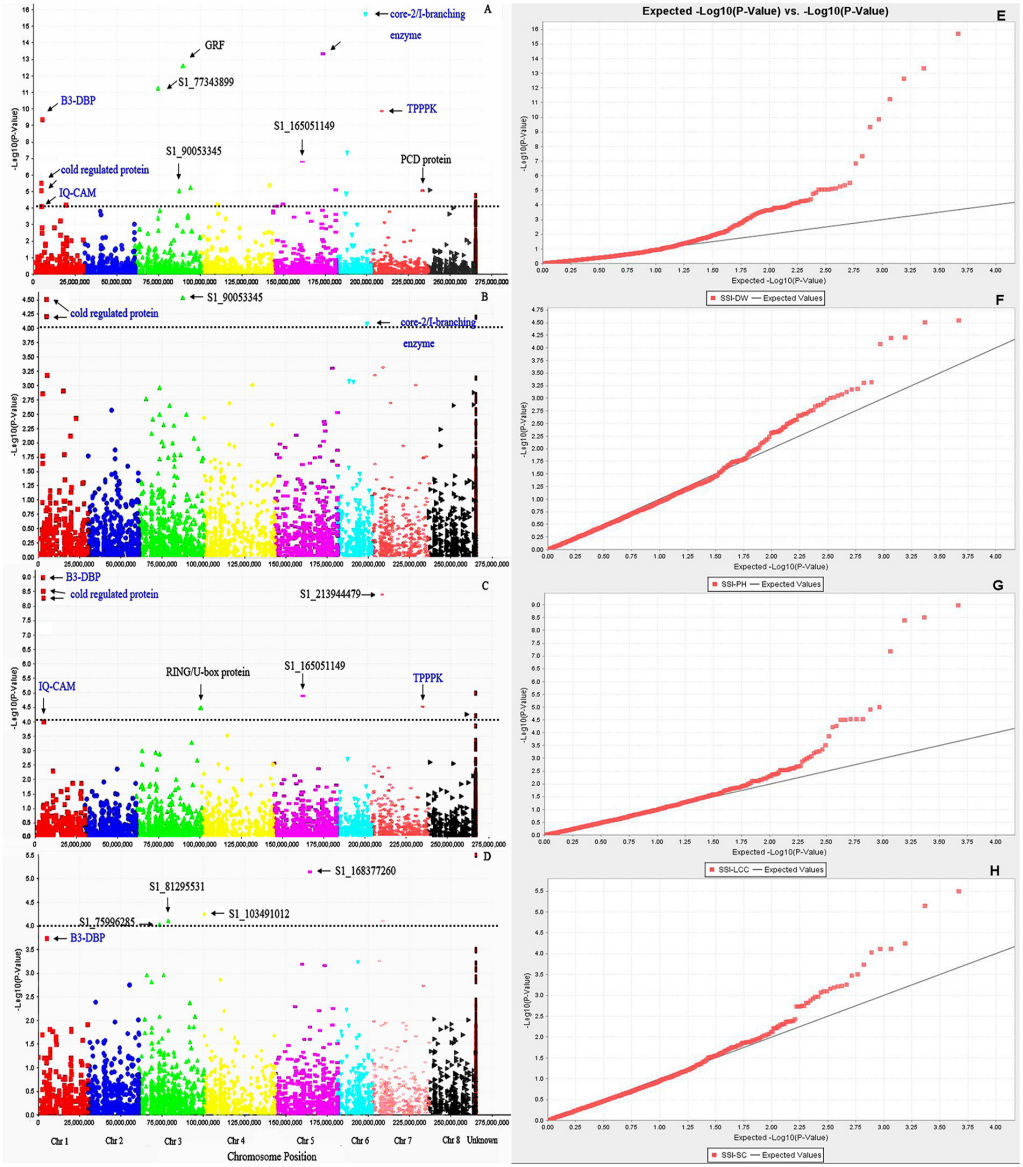
**Manhattan plots of marker –trait association for salt tolerance traits: (A)** SSI-DW, **(B)** SSI-LCC, **(C)** SSI-PH, **(D)** SSI-SC. Significant markers passed a cutoff –log (*P*-value) of 4 were above the dot lines. Putative candidate genes linked to significant markers were labeled and those identified in multiple traits were highlighted by blue color. **(E–H)** represent quantile-quantile (Q-Q) plots from GWAS of SSI-DW, SSI-LCC, SSI-PH, and SSI-SC, respectively. The color curves represent observed *p*-values (log transformed negatives) of marker-trait association. The black line represents expected *p*-values.

We first used phenotypic data without converting to SSI values for GWAS. However, the marker-trait association was very low. The use of SSI values helped in increasing significances of marker-trait association as it is not only measuring the plant response to stress but also considering the genotype variation under non-stress condition. So we choose to use the SSI value in the present analysis. Given that salt concentrations may vary from pot to pot, we added Soil EC as covariate to correct the possible variation in the association. However, there is no difference on the results of the association analysis with or without this cofactor. The marker's *P*-value was used to determine the significance of marker-trait association. The distribution of *P*-values (log transformed negatives) against chromosomal positions for each trait is presented in the manhattan plots (Figures 3A–D), and the observed *P*-values against expected *P*-values are shown in the quantile-quantile (QQ) plots (Figures 3E–H). Using the FDR of 0.05 as described in the Materials and Methods, a total of 42 SNPs were identified to be significantly associated with four traits (Table 3, Figure 3, above dot lines). Most of significant markers (66.67%) were located on Chromosomes 1, 3, 5, and 7. Marker's *R*^2^-values ranged from 0.08 to 0.38 with *P*-values of 1.39E-04 to 1.96E-16 (Table 3). The highest *R*^2^-value (*R*^2^ = 0.38) was found for marker S1_203399656 and it was significantly associated with dry weight under salt stress. Of those identified, 33, 14, 5, and 7 SNPs were significantly associated with dry weight, plant height, leaf chlorophyll content, and stomatal conductance, respectively. Among them, several markers were significantly associated with multiple traits. For example, Marker S1_6017132 and S1_213944479 were significantly associated with dry weight, chlorophyll content and stomatal conductance. Marker S1_5487587 and S1_5487588 located on chromosome 1 were significantly associated with dry weight, chlorophyll content and plant height. Six markers (S1_165051149, S1_238398605, S1_238398606, S1_238398607, S1_288752900, and S1_356690471) showed significant association with chlorophyll content and dry weight. On the same chromosome, three markers (S1_203399656, S1_90053345, and S1_330116900) were significantly associated with dry weight and plant height. The remaining markers were identified for only one trait.

**Table 3:** **Significant SNP markers associated with stress susceptibility indexes (SSI) for measured traits**.

**Trait**			**SSI-DW**			**SSI-PH**			**SSI-LCC**			**SSI-SC**			
**Marker**	**Variant**	**Chr**.	***F***	***P*-value**	***R*^2^**	***F***	***P*-value**	***R*^2^**	***F***	***P*-value**	***R*^2^**	***F***	***P*-value**	***R*^2^**	**Candidate**
S1_203399656	T/A	6	46.32	1.96E-16	0.38	9.98	8.46E-05	0.12	NS	NS	NS	NS	NS	NS	core-2/I-branching enzyme
S1_177465179	G/A	5	37.02	4.50E-14	0.33	NS	NS	NS	NS	NS	NS	NS	NS	NS	FPGS
S1_92290094	T/C	3	35.07	2.41E-13	0.31	NS	NS	NS	NS	NS	NS	NS	NS	NS	GRF
S1_77343899	A/G	3	30.56	5.75E-12	0.28	NS	NS	NS	NS	NS	NS	NS	NS	NS	–
S1_213944479	G/T	7	26.62	1.36E-10	0.27	NS	NS	NS	22.13	3.97E-09	0.23	10.09	7.84E-05	0.12	–
S1_6017132	A/G	1	24.85	4.59E-10	0.25	NS	NS	NS	18.48	6.52E-08	0.25	9.09	1.86E-04	0.11	B3-DBP
S1_192366282	A/G	6	18.98	4.61E-08	0.20	NS	NS	NS				NS	NS	NS	–
S1_165051149	T/C	5	30.06	1.48E-07	0.15	NS	NS	NS	20.3	1.22E-05	0.11	NS	NS	NS	–
S1_5487588	T/G	1	23.17	3.12E-06	0.11	16.8	6.26E-05	0.08	38.91	3.06E-09	0.18	NS	NS	NS	cold regulated protein
S1_5487587	T/G	1	16.26	8.15E-05	0.08	18.24	3.16E-05	0.09	41.49	1.05E-09	0.19	NS	NS	NS	cold regulated protein
S1_144642771	C/T	4	13.21	4.36E-06	0.13	NS	NS	NS	NS	NS	NS	NS	NS	NS	xylose isomerase
S1_96848431	A/T	3	22.04	5.49E-06	0.12	NS	NS	NS	NS	NS	NS	NS	NS	NS	–
S1_185177431	G/A	5	12.75	7.61E-06	0.14	NS	NS	NS	NS	NS	NS	NS	NS	NS	–
S1_245279638	T/A	8	12.62	7.96E-06	0.13	NS	NS	NS	NS	NS	NS	NS	NS	NS	–
S1_238398605	A/T	7	12.42	8.72E-06	0.12	NS	NS	NS	11.05	2.94E-05	0.11	NS	NS	NS	TPPPK
S1_238398606	T/C	7	12.42	8.72E-06	0.12	NS	NS	NS	11.05	2.94E-05	0.11	NS	NS	NS	TPPPK
S1_238398607	C/A	7	12.42	8.72E-06	0.12	NS	NS	NS	11.05	2.94E-05	0.11	NS	NS	NS	TPPPK
S1_90053345	T/C	3	20.92	9.14E-06	0.11	18.48	2.87E-05	0.12	NS	NS	NS	NS	NS	NS	–
S1_191745080	T/G	6	11.98	1.44E-05	0.13	NS	NS	NS	NS	NS	NS	NS	NS	NS	O-glycosyl hydrolase family 17 protein
S1_385914996	A/G	1^*^	11.7	1.78E-05	0.12	NS	NS	NS	NS	NS	NS	NS	NS	NS	cytochrome P450
S1_299186321	T/C	1^*^	10.7	4.05E-05	0.11	NS	NS	NS	NS	NS	NS	NS	NS	NS	3'-5' exonuclease
S1_288752900	T/A	1^*^	10.54	4.70E-05	0.10	NS	NS	NS	12.26	1.02E-05	0.12	NS	NS	NS	IQ-CAM
S1_290085723	T/C	U	17.34	4.87E-05	0.09	NS	NS	NS	NS	NS	NS	NS	NS	NS	–
S1_153571394	T/C	5	10.32	5.63E-05	0.10	NS	NS	NS	NS	NS	NS	NS	NS	NS	polyol/monosaccharide transporter 1
S1_305729693	T/A	7^*^	10.39	5.63E-05	0.11	NS	NS	NS	NS	NS	NS	NS	NS	NS	PCD protein
S1_113265269	T/C	4	16.92	5.86E-05	0.08	NS	NS	NS	NS	NS	NS	NS	NS	NS	–
S1_20159066	T/C	1	10.18	6.91E-05	0.11	NS	NS	NS	NS	NS	NS	NS	NS	NS	syntaxin of plants protein
S1_303117164	C/T	U	10.21	6.97E-05	0.12	NS	NS	NS	NS	NS	NS	NS	NS	NS	E3 ubiquitin-protein ligase ARI7, putative
S1_149685785	C/T	5	16.35	7.72E-05	0.08	NS	NS	NS	NS	NS	NS	NS	NS	NS	–
S1_330116900	C/T	U	9.76	9.52E-05	0.10	10.22	6.32E-05	0.13	NS	NS	NS	NS	NS	NS	–
S1_259280034	G/A	8	9.69	1.01E-04	0.13	NS	NS	NS	NS	NS	NS	NS	NS	NS	–
S1_305729831	C/T	7^*^	9.53	1.18E-04	0.14	NS	NS	NS	NS	NS	NS	NS	NS	NS	PCD protein
S1_356690471	A/T	4^*^	15.25	1.33E-04	0.08	NS	NS	NS	15.15	1.39E-04	0.08	NS	NS	NS	peptide/nitrate transporter
S1_102344252	A/C	3	NS	NS	NS	NS	NS	NS	18.21	3.17E-05	0.09	NS	NS	NS	RING/U-box protein
S1_102344288	T/A	3	NS	NS	NS	NS	NS	NS	18.21	3.17E-05	0.09	NS	NS	NS	RING/U-box protein
S1_267271170	A/G	8	NS	NS	NS	NS	NS	NS	17.21	5.51E-05	0.10	NS	NS	NS	–
S1_290477928	A/G	7^*^	NS	NS	NS	NS	NS	NS	16.82	6.15E-05	0.08	NS	NS	NS	exportin-T-like protein
S1_330575424	T/C	U	NS	NS	NS	NS	NS	NS	NS	NS	NS	13.69	3.19E-06	0.14	–
S1_168377260	G/T	5	NS	NS	NS	NS	NS	NS	NS	NS	NS	21.39	7.08E-06	0.12	–
S1_103491012	T/A	4	NS	NS	NS	NS	NS	NS	NS	NS	NS	17.08	5.63E-05	0.09	–
S1_81295531	T/C	3	NS	NS	NS	NS	NS	NS	NS	NS	NS	16.36	7.70E-05	0.08	–
S1_75996285	C/G	3	NS	NS	NS	NS	NS	NS	NS	NS	NS	9.86	9.31E-05	0.11	–

Same markers identified in different traits were highlighted by same color. Underlined markers with the same first 6 digitals are located at the same locus. Candidate, putative candidate genes linked to the significant markers. FPGS, folylpolyglutamate synthase; GRF, growth-regulating factor; B3-DBP, B3 DNA-binding protein; TPPK, Thiaminepyrophosphokinase; IQ-CAM, IQ calmodulin-binding motif protein; PCD, programmed cell death protein.

“*″ *Unknowns were reassigned to corresponding chromosomes based on the BLAST of the flanking sequence tags against the updated version of M. truncatula genome (Mt4.0 v1)*.

### Assigning significant markers to candidate genes

To assess putative candidate genes underlying marker loci for salt tolerance, we performed a BLAST search using the flanking sequences of the significant SNPs against the *M. truncatula* Mt4.0v1 genome sequences in Phytozome (https://phytozome.jgi.doe.gov/). The BLAST search identified 19 homologs genes to 24 significant markers identified in this study (Figure 3, Table 3). Among them, B3 DNA-binding protein (B3-DBP) showed high homology to marker S1_6017132 on chromosome 1. On the same chromosome, markers S1_5487588 and S1_5487587 at the same locus showed high homology to a cold-regulated protein, and marker S1_288752900 showed homology to IQ calmodulin-binding motif protein (IQ-CAM). On chromosome 3, markers S1_102344252 and S1_102344288 at the same locus showed high homology to RING/U-box protein, and S1_92290094 showed homology to growth-regulating factor (GRF). Marker S1_177465179 on chromosome 5 showed homology to folylpolyglutamate synthase (FPGS). On chromosome 6, markers S1_299186321, S1_191745080, and S1_203399656 showed homology to 3'-5' exonuclease, O-glycosyl hydrolase family 17 protein core-2/I-branching enzyme, respectively. On chromosome 7, Thiaminepyrophosphokinase (TPPPK) showed homology to three markers (S1_238398605, S1_238398606, and S1_238398607) at the same locus. Peptide/nitrate transporter showed homology to S1_356690471 and cytochrome P450 homologs to S1_385914996. Marker S1_305729693 and S1_305729831 at the same locus homolog to a programmed cell death (PCD) protein.

### Linkage disequilibrium analysis of significant markers

**Figure 4:**
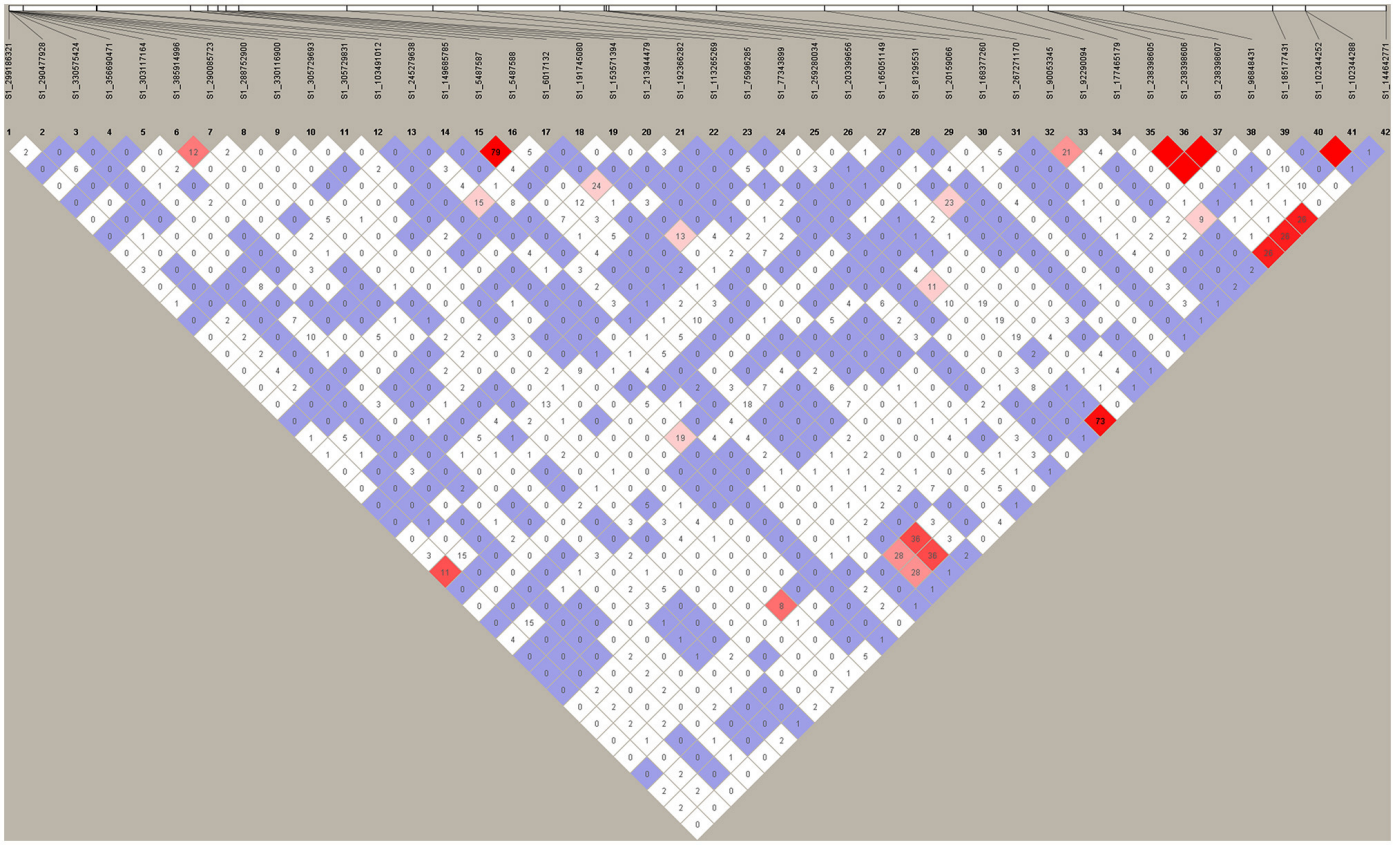
**Linkage Disequilibrium (LD) amongst significant markers**. HAPLOVIEW v.4.2 (http://www.broadinstitute.org/haploview/haploview) pairwise LD values (r^2^^*^100) for 42 SNPs based on genotypes of 198 individuals were used to test whether any of the SNPs significantly associated with salt tolerance were in strong LD with one another. Bright red coloring indicates D' = 1, LOD ≥ 2; Blue coloring indicates D' = 1, LOD < 2; White coloring indicates D' < 1, LOD < 2; Shades of pink/red coloring indicates D' < 1, LOD ≥ 2.

To identify the linkage of markers identified in the present study, we performed linkage disequilibrium (LD) analysis using their marker's data by HAPLOVIEW v.4.2 (http://www.broadinstitute.org/haploview/haploview). The D'- and *r*^2^-values between two markers were calculated and the result is presented in Figure 4. Among 42 markers identified, we observed 4 strong LDs between SNPs S1_102344252 and 102344288, S1_238398605, and S1_238398606, S1_238398606 and S1_238398607, S1_238398605 and S1_238398607, (Figure 4, red prisms). *r*^2^-values between these pairs were 1, suggesting they were tightly linked each other. This was expected as each pair is located at the same locus. Besides the markers with at the same location, high *r*^2^-values were also observed between markers S1_259280034 and S1_144642771 (*r*^2^ = 0.73), and S1_5487587 and S1_5487588 (*r*^2^ = 0.79). The former pair located on different chromosomes (Chromosomes 8 and 4, respectively) while the latter pair is closely located on the same chromosome (chromosome 1). For the remaining SNPs, we did not observe any pair with *r*^2^ > 0.4, suggesting that these loci are likely to contribute to phenotypic variations of the respective traits independently.

## Discussion

### Loci associated with salt tolerance in *Medicago*

Abiotic stresses such as drought and salinity are severely affecting the yield of *Medicago*. Successful application of genomic tools requires both a good biological knowledge and the mechanisms underlying tolerance to these stresses. However, the complexity of the autotetraploid alfalfa (*M. sativa*) genome has hampered this goal. *M. truncatula* has been used as model plant to investigate the genetic bases of nodulation and other important processes such as tolerance to stresses as its small genome and diploid natures (2n = 16) (Dita et al., 2006). Numerous reports have been made on plant responses to salt stress and genes involved in salt tolerance have been identified and characterized in this species (See Rose, 2008 for review). QTLs for salt tolerance have been identified on chromosomes 1 and 8 in *M. truncatula* using recombinant inbred lines derived from a cross between the tolerant and susceptible parents (Arraouadi et al., 2011, 2012). However, limit report has been made on the genetics of the cultivated alfalfa. We have previously reported the identification of genetic loci associated with germination under salt stress in alfalfa (Yu et al., 2016). In the present study, we identified 42 marker loci associated with forage yield and plant growth (Table 3). Compared to the previous study (Yu et al., 2016), more loci and wider distribution on the genome were identified in the present analysis. This was not surprised since more traits were analyzed in the present than in the previous studies. Moreover, the yield-related traits such as dry weight and plant height analyzed in the present study are more complex and are likely polygenic inheritance. There was a trend toward most significant markers on chromosome 1 in both studies. Among them, the B3-DPB gene was consistently identified for all the traits analyzed in present analysis and germination under salt stress in the previous study. Comparable result was also obtained in *M. truncatula* (Arraouadi et al., 2012) where most of loci associated with salt related traits were located on chromosome 1. However, markers identified on chromosomes 5 and 6 in *M. sativa* by the present study were not reported in *M. truncatula* (Arraouadi et al., 2011, 2012).

Of significant markers identified by the present study, eight (S1_213944479, S1_6017132, S1_192366282, S1_238398607, S1_238398606, S1_238398605, S1_288752900, and S1_259280034) were also identified to be associated with germination under salt stress by our previous study (Yu et al., 2016), suggesting that these loci contribute to both germination and plant growth and yield under salt stress. Whereas, the rest were differentially associated with respective traits. Among markers identified in the present study, five (S1_203399656, S1_5487587, S1_5487588, S1_90053345, and S1_330116900) were identified in both dry weight and plant height suggest that they are likely play roles in forage yield as plant height is one of yield components. Additional markers associated with two or more traits identified in the present study are also critical for salt tolerance as all traits evaluated are related to salt tolerance. With further validation, these markers may be useful for maker-assisted breeding to improve salt tolerance in alfalfa.

### Putative candidate genes linked to marker loci for salt tolerance

Several functional genes linked to the lcoi identified in the present study provide putative underlying candidates for involvement in the plants' response to salt stress. For instance, SNP marker S1_177465179 on chromosome 5, linked to folylpolyglutamate synthase (FPGS), which plays a role in lignin biosynthesis in dicot species (Li et al., 2015). Lignin is a phenolic polymer that reinforces the secondary cell wall, confers structural integrity to the plant, aids in water transport and also plays an important role in the plant's responses to various environmental stresses (Holly and Baxter, 2013). A growth-regulating Factor (GRF) was found to be linked to marker S1_92290094 on chromosome 3. Recent studies have shown that GRF was regulated by osa-MIR396c, one member of the miR396 family that mediates plant salt-alkali stress responses in rice (Gao et al., 2010). On chromosome 1, SNP S1_385914996 was linked to cytochrome P450, which has been identified as a salt-responsive gene strongly induced in root after salt stress in *Arabidopsis thaliana* (Ma et al., 2006). Another marker, S1_299186321 on chromosome 1, is linked to 3'-5' exonuclease. It has been reported that Exonuclease transcripts accumulate at high levels in senescing leaves when stress is applied (Tang and Sakamoto, 2011). In addition, a programmed cell death protein (PCD) was found to be linked to markers S1_305729693 and S1_305729831. It has been reported that PCD was identified as a candidate gene responsible for plants to survive biotic (Greenberg and Yao, 2004) and abiotic stress such as salinity and cold stress (Kratsch and Wise, 2000; Huh et al., 2002).

A core-2/I-branching enzyme was found to be linked to marker S1_203399556. Core-2/I-branching beta-1, 6-N-acetylglucosaminyltransferase is a member of Core-2/I-Branching enzyme family that has been shown to be inhibited in response to a long exposure to low temperature in grapevine (*Vitis* spp.) (Kim et al., 2016). Markers S1_102344252 and S1_102344288 on chromosome 3 were linked to a RING/U-box protein. Many plant U-Box genes have been identified as up-regulated in response to salinity conditions (Banzai et al., 2002; Cho et al., 2008).

Cold-regulated protein is a gene linked to S1_5487587 and S1_5487588, which are associated with three traits, SSI-DW, SSI-LCC, and SSI-PH. Cold-regulated protein identified in *M. truncatula* belongs to the family of WRKY transcription factors (TFs) that play a role in biotic stress associated processes (Wang et al., 2006). It has been reported that the expression of WRKY TFs was significantly altered under salt stress in *M. truncatula* (Li et al., 2009). Marker S1_6017132 on chromosome 1, linked to B3-DBP, identified in the present study was also identified as one of the most significant markers in response to salt stress during germination in our previous study (Yu et al., 2016). Additionally, the same gene has been identified by screening a cDNA library induced by salt stress in alfalfa by another group (Jin et al., 2010). The identification of B3-DBP, TPPPK, and IQ-CAM in the present and previous investigations (Yu et al., 2016) suggests that they are likely to play roles in response to salt stress.

In conclusion, in the present study, we identified 42 SNP markers highly significant associated with salt-tolerance traits. They are located on all chromosomes except chromosome 2. Of those identified, 13 were associated with multiple traits. Eight loci identified in the present study were also identified in previous reports. BLAST search revealed that 19 putative candidate genes linked to 24 significant markers. Among them, B3 DNA-binding protein, Thiaminepyrophosphokinase, and IQ calmodulin-binding motif protein were identified among multiple traits in the present and previous (Yu et al., 2016) studies, suggesting that they may play critical roles in plant response to salt stress. Additional markers were associated independently to the respective traits. As different mechanisms exist among different traits, loci conferred to the salt tolerance may via different pathways. Understanding their specific roles of the loci identified in this study required additional investigation such as haplotype analysis of individuals. To this end, we will develop more advanced alfalfa populations for salt tolerance in collaboration with other scientists and will validate these loci in a wide-range of germplasm with known levels of resistance to salt stress. The haplotype analysis will be carried out for validation the markers as described by Patil et al. (2016). Once validated, the haplotypes co-segregated with traits for salt tolerance will be used for developing markers for MAS to improve alfalfa cultivars with enhanced salt tolerance, and the putative candidates underlying these loci can be used for further investigations such as gene cloning and functional characterization.

## Author contributions

Conceived and designed the experiments and reviewed the manuscript: LY. Performed the phenotyping association mapping and writing the manuscript: XL.

### Conflict of interest statement

The authors declare that the research was conducted in the absence of any commercial or financial relationships that could be construed as a potential conflict of interest.

## Supplementary material

Table S1 **The mean SSI values of phenotypic evaluation of traits related to salt stress in the association panel**. SSI-LCC, for leaf relative chlorophyll content; SSI-PH, for plant height; SSI-SC, for stomatal conductance; SSI-RWC, SSI for relative water content; SSI-DW, for dry weight.
